# Lifestyle Adjustment and Mobility-Related Goal Setting After Driving Cessation With People Living With Dementia

**DOI:** 10.1093/geroni/igaa057.927

**Published:** 2020-12-16

**Authors:** Theresa Scott, Jacki Liddle, Elizabeth Beattie, Louise Gustafsson, Geoffrey Mitchell, Donna Rooney, Nancy Pachana

**Affiliations:** 1 The University of Queensland, St Lucia, Queensland, Australia; 2 Queensland University of Technology, Brisbane, Australia; 3 Griffith University, Brisbane, Queensland, Australia; 4 The University of Queensland, Brisbane, Queensland, Australia

## Abstract

Community mobility is an important social determinant of health. For people living with dementia, the forfeiture of a driving licence can signal a loss of independence, limiting access to activities outside of the home. Loss of community connectivity and social participation has a substantial impact on quality of life and may lead to depression and more rapid cognitive decline. This study is focused on a driving cessation intervention that helps people with dementia identify personal goals that are framed around community mobility and adjusting to life without driving. Health professionals work with participants to translate these into specific, practical and achievable outcomes by program end. Participants may nominate more than one goal. This study reports on goal setting and achievement. Using a modified version of the Canadian Occupational Performance Measure it examines pre- to post-intervention achievement of, and satisfaction with, identified goals for 17 participants living with dementia aged 63-93 (M=75.24, 76% male) from regional and metropolitan Australia. Thematic analysis of clinical interviews and field notes highlighted the range of desired goals, and the challenges posed and problem-solving strategies used in setting realistic, non-driving goals. Significant positive improvements were found across a total of 29 goals for (i) performance t(28) = -10.01, p < .000, and (ii) satisfaction, t(28) = -10.32, p < .000. The implications for practice are that supportive goal-setting of personally relevant objectives and valued activities following driving cessation may be effective in lessening some of the negative effects of giving up driving for people with dementia.

